# Obesity Cardiomyopathy in Sudden Cardiac Death: A Distinct Entity? A Comparative Study

**DOI:** 10.1016/j.jacadv.2023.100414

**Published:** 2023-07-28

**Authors:** Joseph Westaby, Chiara Dalle-Carbonare, Irina Chis Ster, Mary N. Sheppard

**Affiliations:** a Cardiovascular Clinical Academic Group and Cardiology Research Section, CRY Centre for Cardiovascular Pathology, Molecular and Clinical Sciences Research Institute, St. George's University of London and St George's University Hospitals NHS Foundation Trust, London, United Kingdom; b Infection and Immunity Research Institute, St George’s University of London, London, United Kingdom

**Keywords:** epicardial fat, hypertrophy, obesity cardiomyopathy, pathology, sudden cardiac death

## Abstract

**Background:**

Obesity cardiomyopathy (OCM) can be associated with sudden cardiac death (SCD) but its pathologic features are not well described.

**Objectives:**

The objective of this study was to characterize the clinical and pathological features of OCM associated with SCD.

**Methods:**

This was a retrospective case control autopsy study. OCM was identified by an increased heart weight (>550 g in males; >450 g in females) in individuals with obesity (body mass index [BMI] ≥30 kg/m^2^) in the absence of other causes. Cases of OCM with SCD were compared to sex and age matched SCD controls with obesity or with normal weight (BMI 18.5-24.9 kg/m^2^) and morphologically normal hearts. Autopsy measures included: heart weight, atrial dimensions, ventricular wall thickness, and epicardial adipose tissue. Fibrosis was assessed microscopically.

**Results:**

Of 6,457 SCD cases, 53 cases of OCM were identified and matched to 106 controls with obesity and 106 normal weight controls. The OCM mean age at death of individuals with OCM was 42 ± 12 with a male predominance (n = 34, 64%). Males died younger than females (40 ± 13 vs 45 ± 10, *P* = 0.036). BMI was increased in OCM cases compared to controls with obesity (42 ± 8 vs 35 ± 5). The average heart weight was 598 ± 93 g in OCM. There were increases in right and left ventricular wall thickness (all *P* < 0.05) in OCM cases compared to controls. Right ventricular epicardial fat was increased in OCM compared to normal weight controls only. Left ventricular fibrosis was identified in 7 (13%) cases.

**Conclusions:**

OCM may be a specific pathological entity associated with SCD. It is most commonly seen in young males with increased BMI.

Obesity has reached epidemic proportions affecting an estimated 13% of the world’s population.^1^ It is defined as a body mass index (BMI) of >30 kg/m^2^. Obesity is a major risk factor for heart failure and sudden cardiac death (SCD)^2^, ^3^, ^4^ and is usually linked to coronary artery disease (CAD), diabetes, or hypertension. Obesity is very common in patients with heart failure with preserved ejection fraction^5^ and has been shown to exacerbate cardiomyopathy.^6^

Obesity is a recognized cause of cardiac enlargement with left ventricular (LV) hypertrophy, diastolic dysfunction, and atrial enlargement^7^, ^8^, ^9^ in both males and females.^10^^,^^11^ Increased heart weight has also been noted in individuals with higher BMIs at post mortem.^12^, ^13^, ^14^ At autopsy, cardiomegaly is seen in individuals with obesity, often explained by coexisting hypertension, CAD, or diabetes.^15^^,^^16^ However, there is growing recognition of individuals with obesity and cardiomegaly where these coexisting conditions are absent, termed obesity cardiomyopathy (OCM).^17^, ^18^, ^19^ This condition can be associated with SCD. In this study, we aim to characterize the clinical and pathological features of OCM associated with SCD by comparing this population to 2 control groups: SCD subjects with obesity or with normal weight and morphologically normal hearts.

## Methods

The study was undertaken at the Cardiac Risk in the Young Centre for Cardiovascular Pathology based at St George’s University of London and is a national referral center for SCD. SCD was defined as an unexpected death occurring instantaneously or within 1 hour of the development of symptoms or if unwitnessed, occurring within 24 hours of last being seen well. Primary care correspondence, clinical notes, coroner’s history, post mortem reports, and family questionnaires were reviewed to obtain circumstances of death and past medical history. Noncardiac causes of death were eliminated by trained autopsy pathologists at the initial autopsy and negative toxicology.

Macroscopic measurements of the ventricular muscle wall and epicardial fat thickness along with the cavity diameters were taken at a midventricular level. The right ventricular outflow tract (RVOT) was measured 10 mm below the pulmonary valve. The left atrium (LA) was measured between the ostia of the left and right superior pulmonary veins and from the atrioventricular junction to the superior surface. The right atrium (RA) was measured from the inferior vena cava ostium to the tip of the appendage and between the ostia of the inferior vena cava and the superior vena cava.

A minimum of 10 blocks are taken for microscopic examination with sections including the RA, LA, RVOT, anterior, lateral and posterior right ventricle (RV), septum, anterior, lateral and posterior LV, the atrioventricular valves, and all 3 coronary arteries. The conduction system was also sampled routinely. More blocks were taken if pathology was identified. Slides were stained with hematoxylin and eosin to examine for fibrosis. A picrosirius red was used to highlight fibrosis, if required.

**Figure 1:**
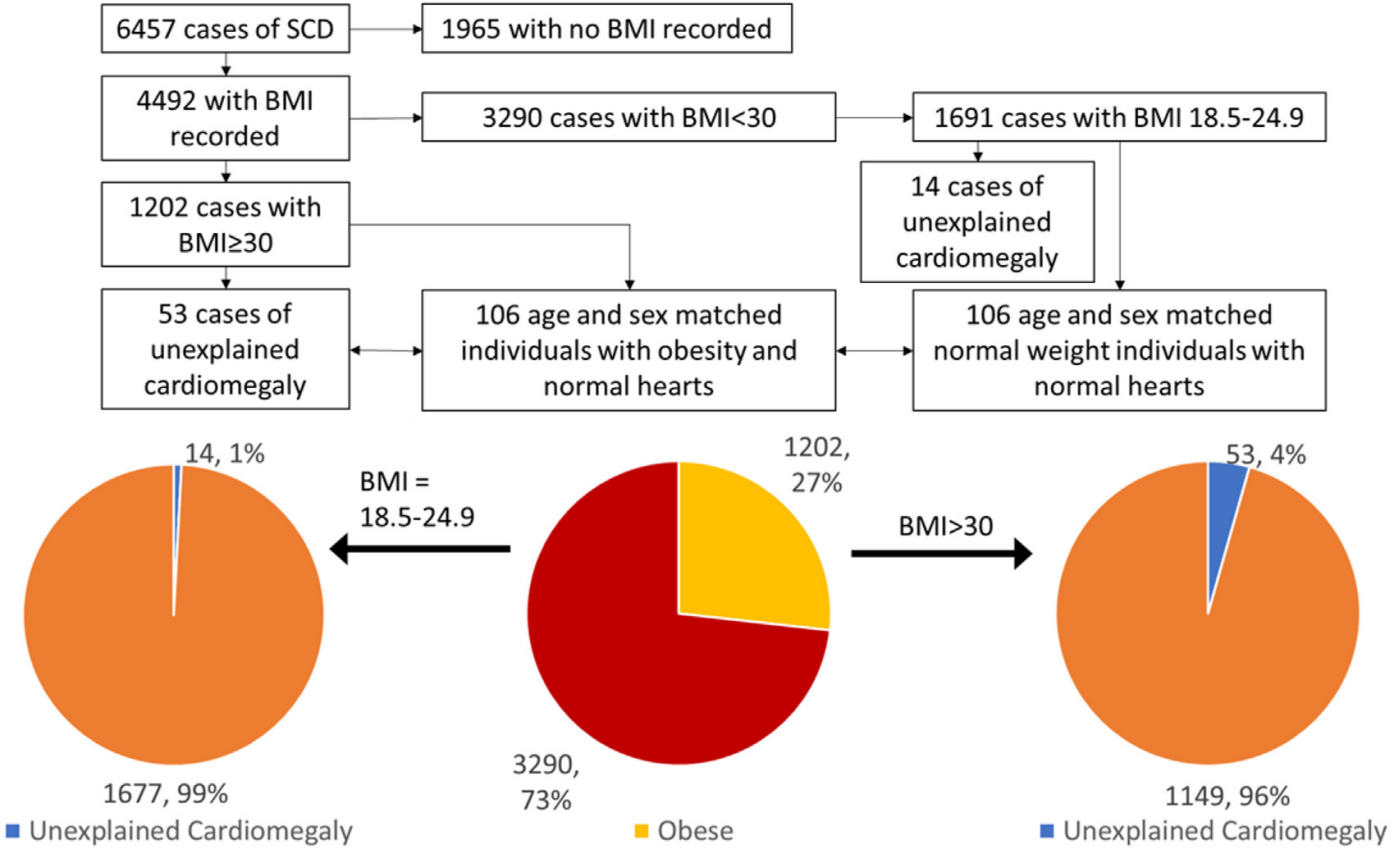
**A Flow Chart of the Study With Pie Charts Illustrating the Frequency of Unexplained Cardiomegaly in Individuals With Obesity and Healthy Weight Individuals** Cases of unexplained cardiomegaly in individuals with obesity were age and sex matched to individuals with obesity and normal weight individuals with a morphologically normal heart. Unexplained cardiomegaly was found in 1% of healthy weight individuals and 4% of individuals with obesity. BMI = body mass index; SCD = sudden cardiac death.

Cases were placed into groups based on BMI. Those with a BMI ≥30 kg/m^2^ were defined as obese and those with a BMI of 18.5 to 24.9 kg/m^2^ were defined as normal weight individuals (Figure 1). Pathological diagnostic criteria used for the classification of unexplained cardiomegaly were increased heart weight above 550 g in males and above 450 g in females in the absence of CAD, hypertension, diabetes, or valvular disease.^20^ Cases with significant CAD (a lumen of <2 mm^2^) were excluded. Hypertrophic cardiomyopathy and infiltrating diseases such as amyloid were excluded on histology. These diagnostic criteria were then applied to individuals with obesity and normal weight group individuals.

Individuals with obesity and cardiomegaly were defined as OCM. Age and sex matched controls with obesity were selected based upon a BMI of >30 kg/m^2^ with a morphologically normal heart weighing <550 g in males and 450 g in females.^20^ Age and sex matched normal weight controls were selected based upon a BMI between 18.5 and 24.9 kg/m^2^ and a morphologically normal heart weighing <550 g in males and 450 g in females. Controls were matched at a 2:1 ratio. In instances where there was more than 1 possible control match to the index OCM case, a random number generator was used for selection.

### Statistics

Categorical and binary data are presented as frequencies (percentages) and continuous data are presented as mean ± SD.

Paired samples *t*-test was used to compare normally distributed continuous variables across 2 matched groups. Wilcoxon matched-pair signed rank test was performed on non-normally distributed or ordinal variables across dependent groups. Given the matching aspect of the data collection, conditional logistic regression was used for associations between cardiomegaly as a binary outcome and various potential explanatory variables measured by odds ratios and their 95% CI. The statistical software package SPSS package 27 (IBM) was utilized to perform these tests. Ethical and research governance approval was granted for this study (10/H0724/38).

## Results

There were 53 cases of otherwise unexplained cardiomegaly from a cohort of 1,202 individuals with obesity, denoted as OCM (Figure 1). There were 14 cases of unexplained cardiomegaly in 1,691 normal weight individuals. The 53 cases of OCM were matched to 106 controls with obesity and 106 normal weight controls. Cardiomegaly was more common in individuals with obesity compared to normal weight individuals (OR: 5.3, 95% CI: 2.9-9.6, *P* < 0.001).

### Clinical characteristics

The mean age at death of 53 individuals with OCM was 42 ± 12 years (vs 36 ± 16 years in the total sudden death cohort) with a male predominance (n = 34, 64% vs n = 4,247, 66% in the total sudden death cohort). Males died younger than females (40 ± 13 vs 45 ± 10, *P* = 0.036). The mean BMI was 42 ± 8 kg/m^2^ (Table 1). The majority of OCM decedents were asymptomatic prior to death (n = 46, 87%), 7 (13%) were symptomatic, 3 (6%) were breathless, 2 (4%) had chest pain, 1 (2%) had syncope, and 1 (2%) had arrhythmia. Obstructive sleep apnea was reported in 4 (8%) individuals.

**Table 1:** Demographics and Body Size Parameters of the 3 Cohorts

	OCM (n = 53)	Controls With Obesity (n = 106)	OCM vs Controls With Obesity *P* Value	Normal Weight Controls (n = 106)	*P* Value	
					OCM vs Normal Weight Controls	Controls With Obesity vs Normal Weight Controls
Age (y)	42 ± 12	41 ± 11	0.624	42 ± 12	0.921	0.470
Male:female	34:19 1.8:1	68:38 1.8:1.0	1.000	68:38 1.8:1.0	1.000	1.000
Height (cm)	178 ± 11	171 ± 10	**0.001**	176 ± 10	0.351	**0.004**
Weight (kg)	132 ± 24	104 ± 18	**<0.001**	70 ± 10	**<0.001**	**<0.001**
BMI (kg/m^2^)	42 ± 8	35 ± 5	**<0.001**	22 ± 2	**<0.001**	**<0.001**
BSA (m^2^)	2.5 ± 0.3	2.2 ± 0.2	**<0.001**	1.8 ± 0.2	**<0.001**	**<0.001**

Values are mean ± SD, n, or ratio unless otherwise indicated. Significant values are given in **bold**.

BMI = body mass index; BSA = body surface area; OCM = obesity cardiomyopathy.

### Circumstances of death

Death occurred at rest or during sleep in 48 (91%) cases, during exertion in 3 (6%) cases, and during a physical altercation in 2 (4%) cases. The physical altercations involved restraint by a security guard and the other involved affray. In these cases, injuries were minor and not deemed sufficient to account for death.

### Autopsy findings in OCM

The mean OCM heart weight was 598 ± 93 g. The heart was heavier in males than females (627 ± 77 g vs 546 ± 99 g, *P* = 0.002) (Supplemental Table 1). The mean heart weight to body weight ratio was 0.47 ± 0.09.

**Figure 2:**
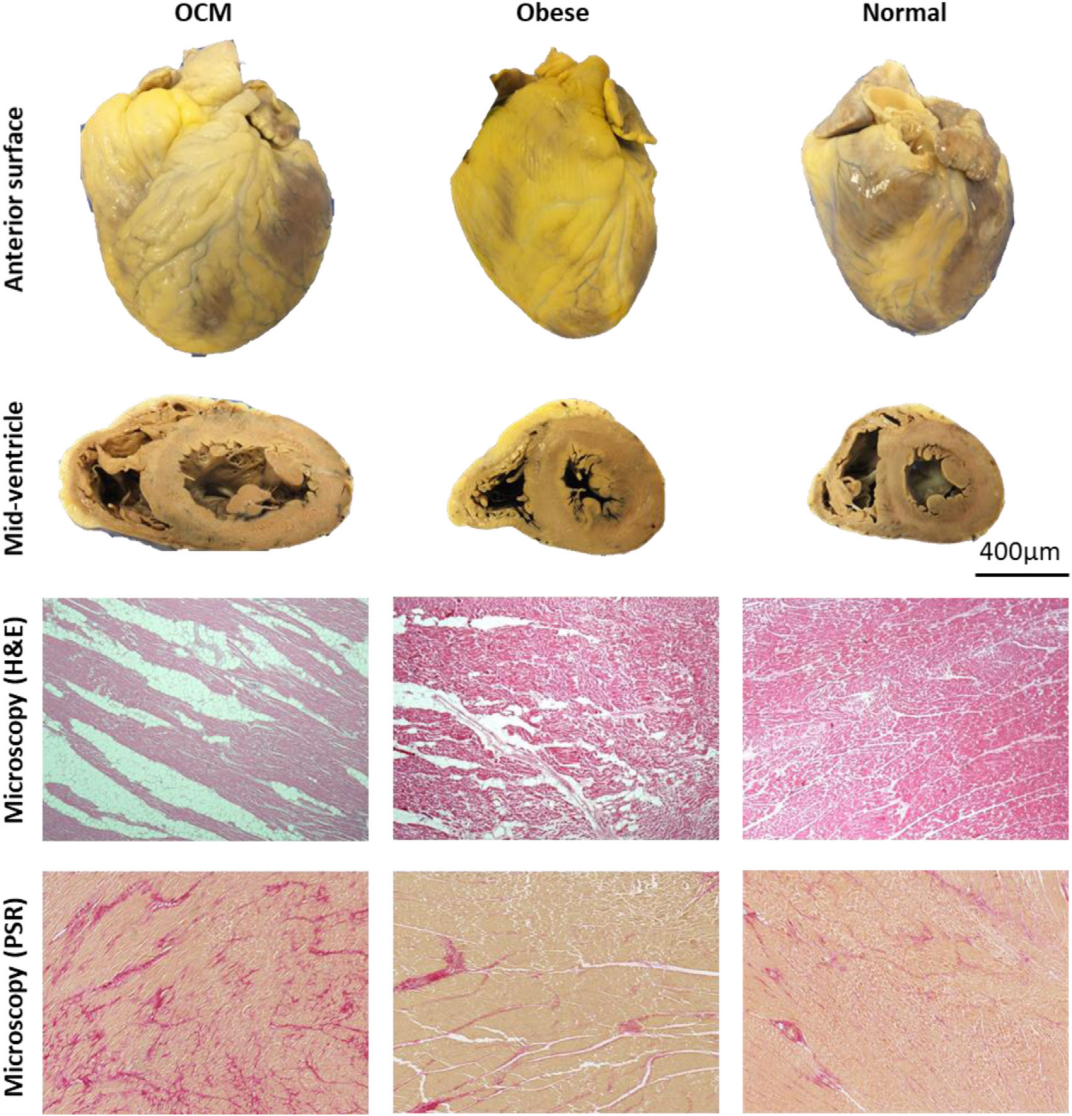
**The Gross and Microscopic Findings in Individuals With Unexplained Cardiomegaly in Obesity, OCM** A greater amount of adipose tissue present on the epicardial surface in the anterior photos. Note the enlargement of the left ventricular wall and cavity dimension on the midventricular slice view. Fatty infiltration may be observed microscopically on hematoxylin and eosin stain. On picrosirius red stain, an increase in fibrosis may be seen. H&E = hematoxylin and eosin; OCM = obesity cardiomyopathy; PSR = picrosirius red stain.

Cavity dimensions, wall thickness, and epicardial fat thicknesses are shown in Table 2. Microscopically all cases showed myocyte hypertrophy and LV fibrosis was present in 7 (13%) cases. The fibrosis was distributed circumferentially in 4 cases, and focally in the anterior wall, the posterior wall, and the septum in 1 case each. The fibrosis was located in the subendocardium in all cases and extended to the midwall in 3 cases. RV fibrosis was not present in any cases (Figure 2).

**Table 2:** Heart Size Parameters for the 3 Groups

	OCM (n = 53)	Controls With Obesity (n = 106)	OCM vs Controls With Obesity *P* Value	Normal Weight Controls (n = 106)	*P* Value	
					OCM vs Normal Weight Controls	Controls With Obesity vs Normal Weight Controls
Heart weight (g)	598 ± 93	400 ± 70	**<0.001**	348 ± 69	**<0.001**	**<0.001**
Right atrial size (cm^2^)	31.3 ± 9.8	21.6 ± 9.4	**<0.001**	25.6 ± 10.1	**0.001**	**0.004**
Left atrial size (cm^2^)	19.9 ± 6.9	22.1 ± 9.1	0.118	16.8 ± 6.5	**0.024**	**<0.001**
Right ventricle						
Cavity diameter (mm)	31.2 ± 8.1	30.1 ± 7.4	0.381	29.4 ± 6.2	0.140	0.445
Anterior wall muscle (mm)	2.8 ± 1.5	2.6 ± 1.0	0.279	2.5 ± 1.0	0.076	0.379
Anterior epicardial fat (mm)	2.8 ± 2.1	1.9 ± 1.8	**0.003**	1.6 ± 1.4	**<0.001**	0.327
Lateral wall muscle (mm)	3.3 ± 1.3	2.8 ± 1.0	**0.011**	2.6 ± 1.1	**<0.001**	0.168
Lateral epicardial fat (mm)	3.5 ± 3.0	3.7 ± 2.5	0.708	3.2 ± 2.8	0.461	0.157
Posterior wall muscle (mm)	4.1 ± 1.1	3.6 ± 0.8	**0.003**	3.4 ± 0.8	**<0.001**	0.088
Posterior epicardial fat (mm)	0.3 ± 0.8	0.2 ± 0.5	0.428	0.2 ± 0.6	0.428	1.000
RVOT wall muscle (mm)	3.9 ± 1.0	3.2 ± 1.1	**0.002**	3.0 ± 1.0	**<0.001**	0.225
RVOT epicardial fat (mm)	1.6 ± 1.3	1.5 ± 1.8	0.539	1.1 ± 1.5	0.063	0.115
Left ventricle						
Cavity diameter (mm)	35.9 ± 6.8	33.6 ± 7.5	0.085	33.3 ± 7.6	0.050	0.762
Septal wall muscle (mm)	15.7 ± 2.8	13.4 ± 4.4	**<0.001**	12.5 ± 2.5	**<0.001**	**0.047**
Anterior wall muscle (mm)	14.6 ± 2.5	12.0 ± 2.1	**<0.001**	11.8 ± 2.3	**<0.001**	0.539
Anterior epicardial fat (mm)	0.8 ± 1.5	1.0 ± 1.5	0.580	1.1 ± 1.8	0.369	0.580
Lateral wall muscle (mm)	14.9 ± 2.8	12.2 ± 2.3	**<0.001**	12.1 ± 2.3	**<0.001**	0.687
Lateral epicardial fat (mm)	0.7 ± 1.4	0.6 ± 1.1	0.641	0.5 ± 1.1	0.220	0.332
Posterior wall muscle (mm)	14.1 ± 2.2	11.7 ± 1.9	**<0.001**	11.6 ± 2.2	**<0.001**	0.667
Posterior epicardial fat (mm)	0.4 ± 1.3	0.3 ± 0.8	0.623	0.3 ± 0.8	0.499	0.814

Values are mean ± SD unless otherwise indicated. Significant values are given in **bold**.

OCM = obesity cardiomyopathy; RVOT = right ventricular outflow tract.

### Controls with obesity

The 106 controls with obesity had a mean age of death of 41 ± 11 years with 68 (64%) males and a mean BMI of 35 ± 5 kg/m^2^. Heart weight was 400 ± 70 g. Macroscopic measurements are provided in Table 2. No myocardial fibrosis was identified (Figure 2).

### Normal weight controls

The 106 normal weight controls had a mean age of death of 42 ± 12 years with 68 (64%) males and a mean BMI of 22 ± 2 kg/m^2^. Heart weight was 348 ± 69 g. Macroscopic measurements are provided in Table 2. No myocardial fibrosis was identified (Figure 2).

### Comparison of cases and controls

The BMI of individuals with OCM were higher than those in both control groups (Table 1). The height of individuals with OCM (178 ± 11 cm) was higher than controls with obesity (171 ± 10 cm) but not normal weight controls (176 ± 10 cm).

**Figure 3:**
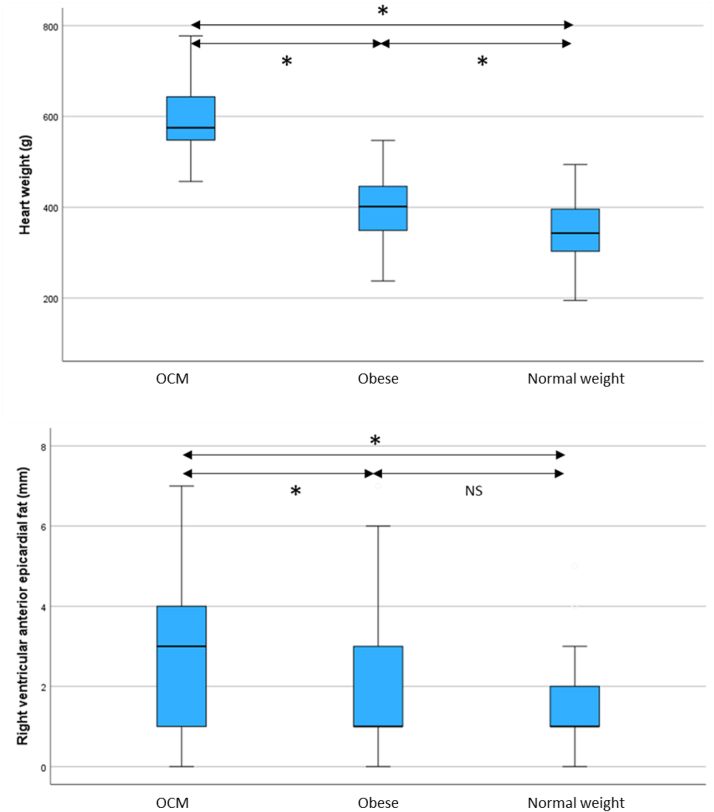
**Boxplot Chart for Heart Parameters** The boxplots show comparisons between the groups for heart weight and right ventricular anterior wall epicardial fat. Hearts from individuals with obesity showed a modest but significant increase in heart weight compared to hearts from normal weight individuals. Obesity cardiomyopathy hearts showed significant increases in right ventricular anterior wall epicardial fat compared to normal controls but not individuals with obesity. ∗*P* < 0.05. NS = not significant; OCM = obesity cardiomyopathy.

**Figure 4:**
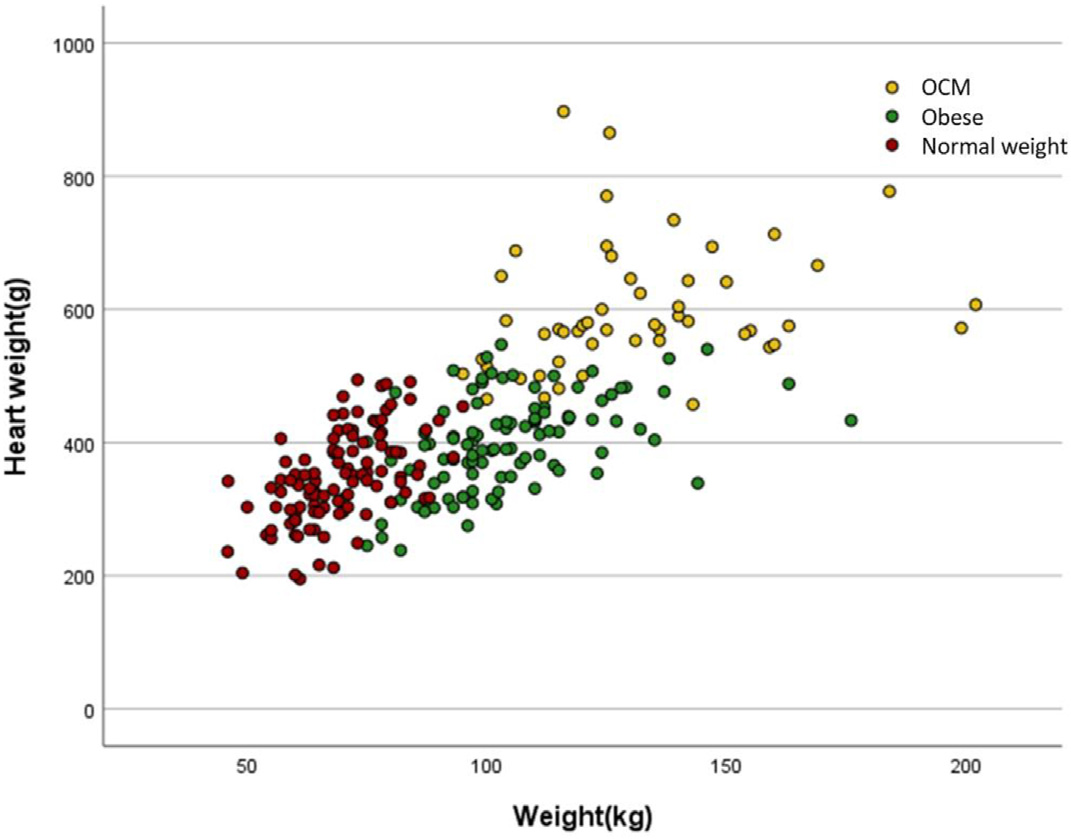
**A Scatterplot of Heart Weight vs Body Weight** Normal weight controls are represented in **red**, controls with obesity are represented in **green** and obesity cardiomyopathy cases are represented in **yellow**. OCM = obesity cardiomyopathy.

The heart weight of individuals with OCM were higher than those in the controls with obesity and the normal weight control group. Hearts from controls with obesity showed a modest but significant increase in weight when compared to normal weight individuals (*P* < 0.001) (Table 2 and Figure 3). The relationship between body weight and heart weight is shown in Figure 4.

The RA cavity dimensions in OCM cases were increased compared to controls with obesity (31.3 ± 9.8 cm^2^ vs 21.6 ± 9.4 cm^2^, *P* < 0.001) and normal weight controls (31.3 ± 9.8 cm^2^ vs 25.6 ± 10.1 cm^2^, *P* = 0.001). The LA cavity dimensions were increased in the OCM cases compared to normal weight controls (19.9 ± 6.9 cm^2^ vs 16.8 ± 6.5 cm^2^, *P* = 0.024).

The RV lateral wall (3.3 ± 1.1 mm vs 2.8 ± 1.0 mm, *P* = 0.011), the RV posterior wall (4.1 ± 1.1 mm vs 3.6 ± 0.8 mm, *P* = 0.003) and the RVOT (3.9 ± 1.0 mm vs 3.2 ± 1.1 mm, *P* = 0.002) showed increases in muscle thickening compared to controls with obesity. The RV lateral wall (3.3 ± 1.1 mm vs 2.5 ± 1.1 mm, *P* < 0.001), the RV posterior wall (4.1 ± 1.1 mm vs 3.4 ± 0.8 mm, *P* < 0.001), and the RVOT (3.9 ± 1.0 mm vs 3.0 ± 1.0 mm, *P* < 0.001) were also increased compared to normal weight controls (Table 2).

The RV anterior epicardial fat was increased in OCM compared to controls with obesity (2.8 ± 2.1 mm vs 1.9 ± 1.8 mm, *P* = 0.003) and normal weight controls (2.8 ± 2.1 mm vs 1.6 ± 1.4 mm, *P* < 0.001) (Table 2, Figure 3).

The LV showed increases in the muscle thickness of the anterior (14.6 ± 2.5 mm vs 12.0 ± 2.1 mm, *P* < 0.001), lateral (14.9 ± 2.8 mm vs 12.2 ± 2.3 mm, *P* < 0.001), and posterior (14.1 ± 2.2 mm vs 11.7 ± 1.9 mm, *P* < 0.001) walls as well as the septum (15.7 ± 2.8 mm vs 13.4 ± 4.4 mm, *P* < 0.001) compared to controls with obesity. The LV showed increases in the muscle thickness of the anterior (14.6 ± 2.5 mm vs 11.8 ± 2.3 mm, *P* < 0.001), lateral (14.9 ± 2.8 mm vs 12.1 ± 2.3 mm, *P* < 0.001), and posterior (14.1 ± 2.2 mm vs 11.6 ± 2.2 mm, *P* < 0.001) walls as well as the septum (15.7 ± 2.8 mm vs 12.5 ± 2.5 mm, *P* < 0.001) compared to normal weight controls (Table 2). See Central Illustration for an overview of the changes.

**Central Illustration:**
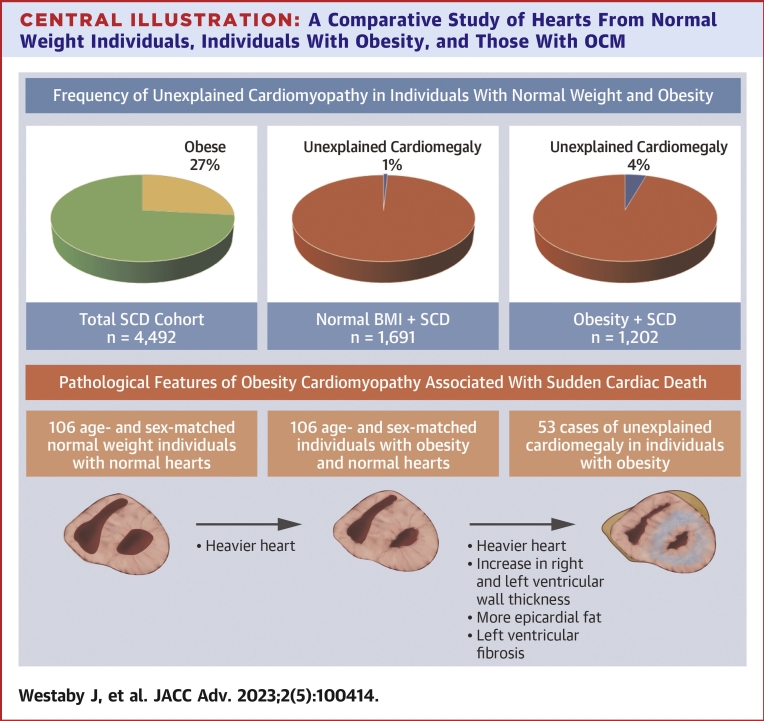
**A Comparative Study of Hearts From Normal Weight Individuals, Individuals With Obesity, and Those With OCM** Cardiomegaly was more common in individuals with obesity than individuals of normal weight. Individuals with obesity have a heavier heart compared to normal weight individuals. Those with obesity cardiomyopathy had even heavier hearts than individuals with obesity. They also showed biventricular hypertrophy and increased epicardial fat compared to obese individuals. Left ventricular fibrosis was seen in a minority of cases. Obesity cardiomyopathy tended to occur in those with a BMI >35 kg/m^2^. Obesity cardiomyopathy may represent a specific entity associated with sudden cardiac death. BMI = body mass index; OCM = obesity cardiomyopathy; SCD = sudden cardiac death.

## Discussion

On autopsy of individuals with SCD, cardiomegaly is 5 times more common in individuals with obesity compared to normal weight individuals. Hearts from subjects with obesity are heavier than heart from normal weight controls. In a proportion of individuals with obesity there is a further increase in heart weight with wall thickening of both the RV and LV. A minority of these individuals show fibrosis. This data supports the case for a new specific pathological entity associated with SCD: OCM. We propose a definition of OCM with SCD as follows: cardiomegaly (>550 g in males and >450 g in females) in individuals with a BMI >30 kg/m^2^ with no history of hypertension or diabetes and no other cardiac disease such as CAD or valve disease at autopsy.

Based on this study, OCM with SCD occurs predominantly in individuals with a BMI of >35 kg/m^2^. SCD victims with obesity are more commonly male and males die at a younger age when compared to females which is also noted clinically.^21^^,^^22^ The pathology is characterized by RV hypertrophy and symmetrical LV hypertrophy in the absence of myocyte disarray or infiltrative disease. Fibrosis is seen in a minority of cases. Biventricular hypertrophy in obesity has been previously noted on imaging in prior studies.^23^ Atrial enlargement was also observed when controls with obesity were compared to normal weight controls suggesting this occurs as a response to obesity and further develops with progression to OCM. The RV hypertrophy seen in OCM may be consequences of increased blood volume and left sided failure or sleep apnea and pulmonary hypertension.^24^ Anterior RV epicardial fat was increased in OCM cases compared to both controls with obesity and normal weight controls suggesting this develops along with cardiac enlargement in obesity. This fat may also contribute to the increased weight of the OCM heart.

### Prior pathology studies

Previous postmortem studies examining OCM have been limited by small numbers,^17^^,^^25^ inclusion of individuals with hypertension, and CAD^18^^,^^25^^,^^26^ or inclusion of hearts weighing <550 g in males and 450 g in females.^17^^,^^26^ Smith and Willis reported on the hearts of 133 individuals with obesity. They found the heart of the individuals with obesity to be heavier than lean individuals, attributed this to increased fat labeling, and termed the condition adiposity of the heart.^26^ They included individuals with heart failure, hypertension, CAD, and other causes of death. In contract, based on the current study, we propose the elevated heart weight is due to muscular thickening rather than adiposity. Amad et al^17^ described 12 subjects with obesity without hypertension or CAD although only 5 showed heart weights meeting our inclusion criteria. Similarly, most of these showed LV hypertrophy and fibrosis was seen in 4 cases. Tavora et al^18^ reported on 484 cases of SCD concluding that cardiomegaly was a cause of SCD and highly associated with obesity; however, they included hypertensives, diabetics, and individuals without obesity. Finally, Roberts and Khan reported on 9 cases of hearts weighing over 1,000 g concluding that obesity should be added as a cause of massive cardiomegaly^25^ but 8 of the cases had hypertension, coupled with diabetes in 5 and the remaining case had Danon disease, so none had pure OCM.

The cause of OCM associated with SCD is not fully understood. Adiposity of the heart^26^ along with cardiac steatosis^27^ and fatty heart^28^ have been used to describe the obese heart. This increased metabolically active epicardial fat found in individuals with obesity and may contribute to the development of cardiomyopathy.^29^ Only a small proportion of cases had fibrosis on microscopy suggesting that SCD in OCM may be mediated through increased ventricular mass. Previous studies have strongly associated increased ventricular mass with SCD.^30^^,^^31^

### Pathological implications

The findings of this study highlight that individuals with obesity who die suddenly have pathological enlargement of the heart in the absence of other causes. The number of cases identified in this study is likely to be an underrepresentation of the incidence of this condition as some cases may be mislabeled as hypertensive heart disease at initial autopsy, despite the absence of a history of hypertension.^15^^,^^32^

### Clinical implications

OCM may represent a specific pathological entity associated with SCD. Public health initiatives to address obesity may be one potential target to decrease SCD risk. For instance, structural heart abnormalities, which occur in individuals with obesity, have been shown to improve following weight loss and bariatric surgery.^33^^,^^34^ The effect of the intervention on SCD risk has yet to be established therefore prospective assessment is warranted.

### Study Limitations

There may be an element of referral bias in the data provided as pathologists may choose to refer more complex cases to the Cardiac Risk in the Young Centre for Cardiovascular Pathology. A small proportion may have had undetected hypertension. Furthermore, all decedents died suddenly and thus the relevance of OCM to the general population with obesity is uncertain. We cannot exclude the existence of electrical abnormalities not detected by pathology or genetics as there is no national electrocardiographic screening program in the United Kingdom.

### Future work

Genetic analysis of cases and follow-up of relatives will allow us to further elucidate any underlying genetic susceptibility to OCM or determine whether this is purely an acquired condition. Furthermore, correlation with clinical phenotype in living patients and association with outcomes is required.

## Conclusions

OCM, defined as cardiomegaly in individuals with obesity without other etiologies of heart disease, may represent a specific pathological entity associated with SCD. In this study, OCM was characterized by RV hypertrophy and symmetrical LV hypertrophy in the absence of myocyte disarray with only a minority showing fibrosis. Its relevance and basis as a marker of risk for SCD now requires assessment in population studies.

PERSPECTIVES **COMPETENCY IN CLINICAL KNOWLEDGE**: Obesity is associated with cardiac enlargement. OCM may represent a specific pathological entity associated with SCD.

**TRANSLATION OUTLOOK**: Public health initiatives to address obesity may be one potential target to decrease SCD risk. Weight loss and bariatric surgery which have been shown to improve cardiac structural abnormalities. The effect of the intervention on SCD risk has yet to be established therefore prospective assessment is warranted.

## Funding support and author disclosures

Cardiac Risk in the Young funds the pathology laboratories and Dr Westaby. The authors have reported that they have no relationships relevant to the contents of this paper to disclose.
