# Supplementary material for: Obesity Cardiomyopathy in Sudden Cardiac Death: A Distinct Entity? A Comparative Study
Source: JACC Adv. 2023 Jul 28;2(5):100414. doi: 10.1016/j.jacadv.2023.100414 (PMC11198605; doi:10.1016/j.jacadv.2023.100414)
Supplement: Supplementary Material [file mmc1.docx]

Supplemental Appendix

# List of investigators

JD WESTABY^a^, C DALLE-CARBONARE^a^, I CHIS STER^b^, MN SHEPPARD^a^

Affiliations:

^a^CRY Cardiovascular Pathology Unit/Cardiovascular Clinical Academic Group and Cardiology Research Centre, Molecular and Clinical Sciences Research Institute, St. George's, University of London and St George's University Hospitals NHS Foundation Trust, London, UK

^b^Infection and Immunity Research Institute, St George’s University of London, London, UK

# Supplemental Methods

Autopsy

Autopsies were performed according to and adhering to the standards of the Royal College of Pathologists of the United Kingdom (https://www.rcpath.org/profession/guidelines/autopsy-guidelines-series.html).

Cardiac dissection and examination

The heart is weighed following the removal of any blood clot. The coronary arteries are sliced at 2-3mm intervals to look for evidence of coronary artery disease. In cases with stenosis, a 2mm probe is used to assess the significance. The right atrium is opened between the inferior vena cava and the superior vena cava and the inferior vena cava and the tip of the atrial appendage. The left atrium is opened between the superior pulmonary veins and between the left superior pulmonary vein and the tip of the atrial appendage. The cavities and appendages are inspected for thrombi. The fossa ovalis is assessed for patency. The atrioventricular valves are inspected from the atrial aspect and assessed for patency. The heart is sliced transversely from the apex to the mid ventricle at 5mm intervals. The presence or absence of fibrosis, infarction, or fat in the myocardium is commented on. The atrioventricular chambers are opened laterally through the atrioventricular valves allowing visualisation of the four chambers and the atrioventricular valves. The semilunar valves are assessed for patency from the ventricles. The right ventricular outflow tract is opened through the pulmonary valve anteriorly. The left ventricular outflow tract is opened through the anterior leaflet of the mitral valve. The ostia of the coronary arteries are inspected to examine for anomalous origin.

Cardiac sections

Blocks are taken for histology routinely from the anterior, lateral and posterior right ventricle, the right ventricular outflow tract, the anterior, lateral and posterior LV as well as the interventricular septum (IVS). Sections of coronary artery, atria and conduction tissue are also sampled routinely. Normally, 10 sections are examined though more blocks will be taken if specific pathology is found. Significant stenosis or thrombosis of the coronary arteries is serially sectioned for histological analysis if observed. Following processing of the blocks, the slides are stained with hematoxylin and eosin. A picrosirius red is used to highlight fibrosis, if required.

Histological analysis

*Hypertrophic cardiomyopathy*

Hypertrophic cardiomyopathy is diagnosed by assessment of the left ventricle for myocyte disarray. A diagnosis is given when myocyte disarray is present in over 20% of two cardiac sections. Myocyte disarray is not assessed at the septal free wall junctions or in the subendocardium, trabeculae or around vessels as this is considered normal. Myocyte disarray also occurs around areas of fibrosis in any condition and therefore this is also discounted. In cases with clear myocyte disarray not reaching these criteria further blocks are taken for examination.

*Amyloid*

Amyloid was diagnosed by the detection of eosinophilic amorphous material within the vessels and interstitium. A congo red was done to confirm the diagnosis and the material was examined for birefringence under polarised light.

*Myocarditis*

A diagnosis of myocarditis was made on the basis of inflammation present in at least 20% of two cardiac sections with associated myocyte necrosis. In cases where inflammation is present not reaching these criteria further blocks are taken for analysis.

*Sarcoidosis*

A diagnosis of sarcoidosis is made on the basis of non-caseating granulomas in the present in the myocardium. Periodic acid–Schiff–diastase, Grocott and Ziehl–Neelsen stains are performed to exclude alternate aetiology.

Supplemental Table 1: Heart size parameter for males and females with obesity cardiomyopathy

| Variable | Unit | Summary statistic | Male (n=34) | Female (n=19) | Univariable p value |
| --- | --- | --- | --- | --- | --- |
| Heart weight | g | Mean±SD | 627±77 | 546±99 | **0.002** |
| **Atria** |  |  |  |  |  |
| Right atrial size | cm^2^ | Mean±SD | 31.2±10.0 | 31.4±9.7 | 0.959 |
| Left atrial size | cm^2^ | Mean±SD | 20.5±7.8 | 19.0±5.3 | 0.502 |
| **Right ventricle** |  |  |  |  |  |
| Cavity diameter | mm | Mean±SD | 32.7±8.5 | 28.7±6.8 | 0.107 |
| Anterior wall muscle | mm | Mean±SD | 3.1±1.6 | 2.4±0.9 | 0.107 |
| Anterior epicardial fat | mm | Mean±SD | 2.5±2.2 | 3.3±1.8 | 0.197 |
| Lateral wall muscle | mm | Mean±SD | 3.3±1.1 | 3.2±1.6 | 0.754 |
| Lateral epicardial fat | mm | Mean±SD | 3.5±3.0 | 3.6±3.2 | 0.896 |
| Posterior wall muscle | mm | Mean±SD | 4.0±0.9 | 4.2±1.5 | 0.561 |
| Posterior epicardial fat | mm | Mean±SD | 0.1±0.4 | 0.5±1.3 | 0.192 |
| RVOT wall muscle | mm | Mean±SD | 3.8±0.7 | 4.0±1.4 | 0.626 |
| RVOT epicardial fat | mm | Mean±SD | 1.5±1.4 | 1.9±1.1 | 0.394 |
| **Left ventricle** |  |  |  |  |  |
| Cavity diameter | mm | Mean±SD | 35.7±6.3 | 36.2±7.8 | 0.820 |
| Septal wall muscle | mm | Mean±SD | 15.9±2.6 | 15.5±3.1 | 0.618 |
| Anterior wall muscle | mm | Mean±SD | 14.7±2.1 | 14.5±3.0 | 0.823 |
| Anterior epicardial fat | mm | Mean±SD | 0.9±1.6 | 0.6±1.4 | 0.542 |
| Lateral wall muscle | mm | Mean±SD | 14.9±2.7 | 15.1±3.0 | 0.823 |
| Lateral epicardial fat | mm | Mean±SD | 0.7±1.6 | 0.7±1.2 | 0.950 |
| Posterior wall muscle | mm | Mean±SD | 14.3±2.0 | 13.7±2.5 | 0.407 |
| Posterior epicardial fat | mm | Mean±SD | 0.3±1.0 | 0.4±1.7 | 0.842 |

The heart size parameters for males and females with obesity cardiomyopathy (OCM). The table shows the heart weight along with atrial and ventricular measurements for both muscular wall and epicardial adipose tissue. RVOT, right ventricular outflow tract.
